# In Silico Comparison of Photon Versus Proton Based Stereotactic Body Radiotherapy With Increasing Maximum Peak Dose for Treatment of Primary Renal Cell Carcinoma

**DOI:** 10.1016/j.ijpt.2025.101210

**Published:** 2025-10-25

**Authors:** Sherif G. Shaaban, Hao Chen, Anh Tran, Aditya Halthore, Rachit Kumar, Michael Goldstein, Daniel Song, Stephen Greco, Heng Li, Curtiland Deville

**Affiliations:** 1 Department of Radiation Oncology and Molecular Radiation Sciences, Johns Hopkins University School of Medicine, Baltimore, MD, USA; 2 Department of Radiation Oncology, University of South Florida Healthcare, Tampa, FL, USA

**Keywords:** Proton therapy, Renal cell carcinoma, SBRT

## Abstract

**Purpose:**

Photon based Stereotactic Body Radiotherapy (PH-SBRT) has emerged as a promising option for treatment of primary renal cell carcinoma (RCC), however is associated with decrements in long-term kidney function and toxicity to adjacent organs at risk (OARs).

**Materials and Methods:**

We compared proton (PT) based SBRT versus PH-SBRT regarding target coverage, OARs, and maximum peak doses of 125% and 142%. All variables were compared using student *t*-tests.

**Results:**

Target coverage was comparable between plans for gross tumor volume (GTV) and clinical target volume (CTV). OARs were greatly spared by PT-SBRT including small bowel; V5 Gy to V25 Gy and maximum dose (10.47, 20.22, *P* = .02), large bowel; V5 Gy to V25 Gy and maximum dose (16.16, 23.90, *P* = .03), all volumes of uninvolved ipsilateral kidney and maximum dose to heart, lungs, esophagus, stomach, duodenum, ipsilateral ureter, and spinal canal. The ratio of ipsilateral kidney V50%/Vtot (volume receiving 50% of the dose/total volume), which is correlated with glomerular filtration rate loss in prospective trials, was significantly lower with PT-SBRT for both maximum dose of 125% (25.41, 19.97, *P* < .01) and 142% (23.33, 19.18, *P* < .01). Integral dose was significantly lower with protons. PT-SBRT with higher peak dose allowed for additional sparing of OARs: stomach V 10 Gy, liver V 25 Gy, large bowel V 30 Gy, V 35 Gy, ipsilateral kidney cortex.

**Conclusion:**

PT-SBRT improved target coverage while reducing dose to the involved kidney and adjacent OARs. Protons allow delivery of maximum peak dose escalated plans without exceeding the normal tissues limit and have the potential to better preserve renal function. Prospective studies are warranted to validate these findings and potential clinical benefits.

## Highlights

• There is a growing interest in using proton therapy in RCC.
• Protons may be advantageous for poor kidney function or tumors near organs at risk.
• PT-SBRT improved coverage and ipsilateral kidney V50%/Vtot, correlated previously with GFR loss.
• Protons allow for maximum peak dose escalated plans and potentially better preserve renal function.

## Introduction

Kidney cancer represents 4% of all new cancer cases in the United States (US).^1^ There has been a consistent increase in the incidence of kidney cancer diagnosis over recent decades that largely attributed to more frequent use of routine imaging studies for unrelated medical reasons. In 2025, renal cell carcinoma (RCC) accounted for 80 980 new cases and 14 510 deaths.^1^ The incidence generally increases with age, peaking in people aged 65 years or older.^1^

Patients with localized diseased can be considered for active surveillance, ablative approaches including cryotherapy, microwave and radiofrequency ablation and surgical resection (partial or radical nephrectomy).^2^ Stereotactic body radiation therapy (SBRT) represents a more recent option for patients who are unsuited for surgery or ablative procedures due to medical comorbidities or other technical limitations.^3^

RCC has long been considered to be less radiosensitive tumor^4^ relative to other tumors. In vitro studies have shown that the alpha/beta (α/β) ratio of RCC is low between 2.6 and 6.92 Gy highlighting that this relative radioresistance can be overcome by using higher dose per fraction.^5^ If assuming an α/β ratio of 3, a biologically effective dose (BED) of BED3 > 225 Gy may achieve optimal rates of control.^6^

Therefore, SBRT has become increasingly popular for treatment of primary RCC.^7^ SBRT uses precisely delivered high doses of radiation and offers several advantages including its nan-invasive nature, short treatment duration and favorable toxicity profile.^7^ Multiple retrospective series have shown that SBRT is safe and effective treatment for primary RCC with excellent local control (95–98%), acceptable side effect profile and modest impact on kidney function decline.^8^, ^9^, ^10^

Recently FASTRACK II, the first multicenter prospective phase II study, was published by Siva and colleagues.^9^ The trial included 70 patients with localized (T1-T2a) RCC and received either 26 Gy single fraction (BED3 = 286 Gy) for smaller tumors or 42 Gy in 3 fractions (BED3 = 268 Gy) for tumors larger than 4 cm. They reported a maximum peak dose range from 125–143%. The local control rate was 100% with a mean eGFR loss of 10.8 mL/min per 1.73 m^2^ (8.5–13.1) at 1 year and by 14.6 mL/min per 1.73 m^2^ (12.1–17.1) at 2 years after treatment.^9^ They found that the ratio of ipsilateral kidney V50%/Vtot (volume receiving 50% of the dose/total volume) with glomerular filtration rate (GFR) loss.

Compared to photons, proton therapy has several advantages, including a favorable dose-depth profile (Bragg peak), a higher linear energy transfer (LET), and a higher relative biological effectiveness (RBE).^11^ These characteristics allow protons to spare more healthy tissue and create more conformal dose distribution to provide the potential to treat challenging tumors, in terms of location (deep seated or critically located), radio-resistance, or a highly aggressive nature.^11^

Despite the existing evidence supporting the use of photon based SBRT (PH-SBRT) for treatment of primary RCC, the dosimetric comparisons for SBRT treatment are still lacking. In this study, we compared proton based SBRT (PT-SBRT) versus conventional, photon-based SBRT (PH-SBRT) for primary RCC regarding target coverage, organ at risk (OAR) sparing, plan conformity, and dose heterogeneity with maximum target peak doses of 125% and 142%.

## Materials and methods

Following institutional review board approval, our institutional database was queried to identify 20 patients treated from 10/2016 to 12/2024 with primary RCC previously treated with PH- or PT-SBRT. Patients with distant metastases were excluded from analysis. Table 1 shows the tumor characteristics of the study cohort.

**Table 1:** Baseline tumor characteristics of the study cohort (*N* = 20 patients).

**Characteristic**	**No. (%)**
Laterality	Left (7, 35%) Right (13, 65%)
Initial Tumor Size	
≤4 cm 4−7 cm >7 cm	4 (20%) 9 (45%) 7 (35%)
Exophytic/endophytic	Exophytic (17, 85%) Endophytic (3, 15%)

### Simulation

Patients were simulated in the supine position with arms above their head using vac loc for immobilization. CT slice thickness was typically 1–2 mm. 4-D CT scanning was used for assessment of target motion. If the target motion exceeded 1.0 cm in the craniocaudal direction, Active Breathing Control (ABC) with breath hold was utilized for motion management. Treatment planning scans were fused to the diagnostic IV contrast CT of the abdomen and/or magnetic resonance imaging when available.

### Volume delineation

All target and OAR contours were delineated by an attending radiation oncologist in the RayStation treatment planning system (RaySearch Laboratories, Stockholm, Sweden). The gross tumor volume (GTV) was defined as all areas of gross tumor seen on imaging. The iGTV included gross tumor in all phases of breathing, including maximal intensity projection, end inspiration, and end expiration. The clinical target volume (CTV) was created equal to the iGTV (with no expansion). Planning target volumes (PTVs) were generated, for the purposes of photon planning and robust optimization of proton planning, using CTV plus 5 mm expansion in the craniocaudal direction and 3 mm in the lateral direction.

Contoured OARs included the lungs, esophagus, heart, liver, stomach, duodenum, small bowel, large bowel, ipsilateral kidney-CTV, contralateral kidney, spinal canal, and body (generally the scanned body from diaphragm through pelvis), and body-CTV.

### Treatment planning

The same prescription dose and planning parameters of FASTRACK II study were used, specifically 42 Gy in 3 fractions of 14 Gy and maximum dose between 125–143%. The coverage parameters were that 99% of the GTV and CTV were covered by 100% of the dose, while 95% of the PTV was covered by 100% of the prescription dose. When OARs could not be met while achieving this level of coverage, an alternative was 90% coverage of the PTV with 100% of the dose. Therefore, photon and proton plans with maximum dose (D max) of 125% and 142% were created separately for each patient with predefined GTV, CTV, PTV, and OAR constraints as noted in Table 2.

**Table 2:** Organ at risk (OAR) constraints.

**Organ**	**Parameter**	**Dose/Fractionation**
		**42Gy/3Fx**
Spinal Canal	Maximum Dose	1.5 cc < 13 Gy
Esophagus	Maximum Dose	1.5 cc < 18 Gy
Heart	Maximum Dose	1.5 cc < 24 Gy
Lung	Maximum Dose	1000 cc < 11 Gy
Skin	Maximum Dose	1.5 cc < 30 Gy
Stomach	Maximum Dose	1.5 cc < 22.5 Gy
Duodenum	Maximum Dose	1.5 cc < 15 Gy
Small Bowel	Maximum Dose	30 cc ≤ 12.5 Gy
Large Bowel	Maximum Dose	20 cc < 24 Gy
Ureter	Maximum Dose	40 Gy
Ipsilateral Renal Hilum	Maximum Dose	ALARA^a^
Ipsilateral Renal Cortex	Maximum Dose	ALARA
Ipsilateral Kidney	Maximum Dose	ALARA, Minimize volume of high dose regions (>50% isodose)
Contralateral Kidney	V10 Gy	≤33%
Liver	Mean dose, Maximum volume	D700 cc ≤ 21 Gy

a ALARA: as low as reasonably achievable.

**Figure 1:**
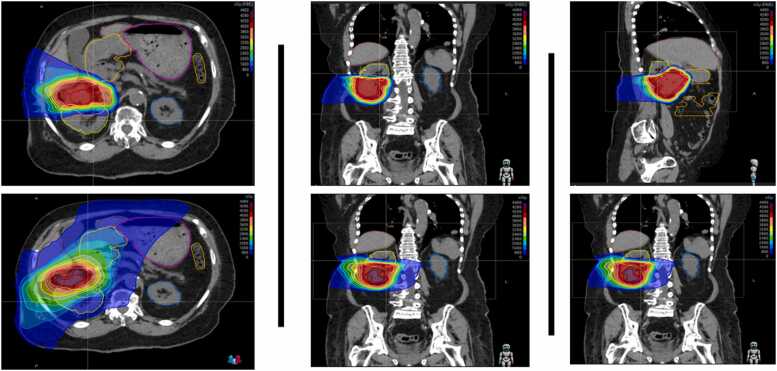
Representative color wash images in axial (left), coronal (middle), and sagittal (right) planes comparing protons (top row) versus photon (bottom row) based SBRT dose deposition for primary RCC. As shown, the target receives similar coverage in the two planes, with greater sparing of nearby structures with protons including the healthy part of the kidneys, stomach, bowel and liver. Abbreviations: RCC, renal cell carcinoma; SBRT, stereotactic body radiotherapy.

Photon plans were generated using volumetric modulated arc therapy (VMAT), consisting of six 360-degree arcs with 6 collimator rotation angles. Proton plans were created using 4–5 lateral oblique and/or posterior oblique beams with proton beam angles selected to avoid the couch edge and OARs. Proton therapy doses were calculated using a radiobiological effective (RBE) dose of 1.1. An example of the dose-color-wash distribution of each plan can be seen in Figure 1.

All plans were generated using the RayStation treatment planning system. The proton plans consisted of intensity modulated proton therapy using pencil beam scanning with discrete spot scanning. Inverse optimization was used to generate appropriate dose distribution with a pre-specified weighting of target coverage and OAR sparing using modulation of beam spot location, energy, and weight. Plans were optimized to deliver a predominantly uniform dose from each field (single-field optimization; SFO) prioritizing sparing of the uninvolved kidneys, liver, bowel and the spinal canal. All proton plan optimization combined expansion and robust optimization with 3.5% range uncertainty and 0.5 cm setup uncertainty. The conformity index displayed in RayStation was used to assess how well the radiation treatment plan conforms to the target volume, calculated using the Paddick formula.

### Statistical analysis

The volumetric percentage of GTV, CTV, PTV, liver, ipsilateral kidney-CTV, ipsilateral kidney cortex, ipsilateral kidney medulla, contralateral kidney, lungs, small bowel, and large bowel along the entire dose volume histogram (DVH) were evaluated. Comparative maximum doses to the heart, esophagus, stomach, duodenum, spinal canal, ipsilateral ureter, skin and body were assessed. Integral dose to Body-CTV was also captured. Student *t*-tests were used to compare the plans, with *P* < .050 considered statistically significant. All statistical analyses were conducted using Matlab Version R2023a (MathWorks, Natick, MA, USA) and Excel Version 16.74 (Microsoft, Redmond, WA, USA).

## Results

### Assessment of 42 Gy in three fractions of 14 Gy with Dmax 125%

**Figure 2:**
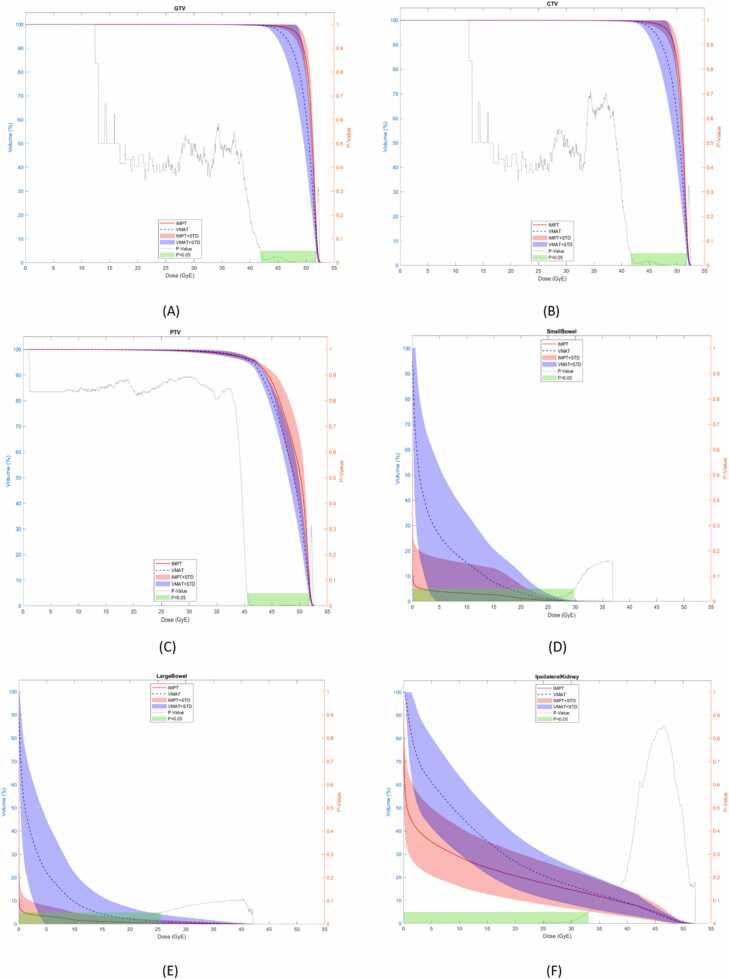
(A–F) (maximum dose 125%): Doses to the targets and organs at risk (OAR) shown over the entire dose–volume histogram comparing intensity-modulated proton therapy (IMPT—red) and Volumetric Modulated Arc Therapy (VMAT—blue), including GTV (A), CTV (B), PTV (C) small bowel (D), large bowel (E), ipsilateral kidney – CTV (F). Abbreviations: CTV, clinical target volume; GTV, gross tumor volume; IMPT, intensity modulated proton therapy; PTV, planning target volume.

Table 3 and Supplementary Table 1 (S1) provide comparative mean values for target coverage and doses to OARs for PH- and PT-SBRT plans. Comparative DVHs for targets and OARs are shown in Figure 2 and Supplementary Figure 1 (S2). Overall the target goals and OAR constraints were met by both photon and proton plans.

**Table 3:** The main dosimetric endpoints of target coverage and OAR doses comparing proton based SBRT (PT-SBRT) and photon based SBRT (PH-SBRT).

Target/OAR	Dosimetric endpoint	Maximum dose 125%			Maximum dose 142%		
		PT-SBRT	PH-SBRT		PT-SBRT	PH-SBRT	
		Mean±SD	Mean±SD	*P*-value	Mean±SD	Mean±SD	*P*-value
GTV	V100% > 99% (%)	99.74±0.32	99.70±0.40	.24	99.80±0.30	99.63±0.40	.09
CTV	V100% > 99% (%)	99.70±0.35	99.62±0.40	.23	99.72±0.33	99.63±0.40	.20
PTV	V100% > 95% (%) V100% > 90% (%)	95.43±0.90 95.43±0.90	94.13±1.81 94.13±1.81	.00^a^ .00^a^	95.54±1.10 95.54±1.10	93.80±1.80 93.80±1.80	.00^a^ .00^a^
PTV	Conformity Index (CI)	0.90±0.04	0.94±0.04	.01^a^	0.90±0.05	0.95±0.03	.01^a^
Heart	Max Dose (GyE)	0.01±0.02	0.10±2.18	.02^a^	0.01±0.02	0.10±2.18	.02^a^
Lungs	Max Dose (GyE)	0.00±0.01	0.18±0.40	.02^a^	0.00±0.01	0.14±0.30	.01^a^
Esophagus	Max Dose (GyE)	0.00±0.02	1.01±2.11	.01^a^	0.00±0.02	1.01±2.11	.01^a^
Stomach	Max Dose (GyE)	1.72±7.62	12.22±10.90	.00^a^	1.70±7.53	8.13±8.65	.00^a^
Duodenum	Max Dose (GyE)	6.15±9.87	12.22±10.90	.02^a^	5.70±9.08	11.70±10.28	.01^a^
Liver	D700cc ≤ 21 (GyE)	0.16±0.40	0.70±0.90	.01^a^	1.61±3.80	6.65±8.11	.01^a^
Small Bowel	Max Dose (GyE)	10.47±13.64	20.22±12.90	.00^a^	10.13±13.50	20.07±11.61	.00^a^
Large Bowel	Max Dose (GyE)	16.16±15.50	23.90±11.54	.03^a^	16.03±15.40	24.23±11.80	.01^a^
Ipsilateral Kidney - CTV	Max Dose (GyE)	0.90±11.30	14.61±0.72	.02^a^	0.81±11.20	12.44±0.74	.04^a^
Ipsilateral Kidney Cortex	Max Dose (GyE)	13.25±14.18	19.70±11.80	.09	12.70±14.00	17.40±13.14	.00^a^
Ipsilateral Kidney Medulla	Max Dose (GyE)	13.57±12.40	18.06±0.91	.13	12.60±12.00	15.30±0.93	.20
Ipsilateral Kidney	V50%/Vtot	25.41±11.04	19.97±9.08	.00^a^	23.33±10.18	19.18±9.33	.00^a^
Contralateral Kidney	Max Dose (GyE)	0.00±0.10	1.90±1.50	.16	0.00±0.10	2.00±1.60	.16
Ipsilateral Ureter	Max Dose (GyE)	15.54±19.70	27.81±16.55	.04^a^	15.40±18.50	24.42±18.32	.04^a^
Skin	Max Dose (GyE)	16.20±5.75	16.65±8.22	.15	17.22±6.01	17.00±8.09	.40
Spinal Canal	Max Dose (GyE)	2.21±4.60	9.00±4.70	.00^a^	2.30±4.71	9.20±4.75	.00^a^
Body	Max Dose (GyE)	52.51±0.20	52.35±0.08	.00^a^	59.30±1.62	59.15±0.90	.01^a^
Body - CTV	Max Dose (GyE)	0.06±0.70	1.70±1.42	.00^a^	0.61±0.70	16.70±1.35	.00^a^

**Abbreviations:** cc, cubic centimeter; CI, Conformity Index; CTV, clinical target volume; GTV, gross tumor volume; GyE, radiobiological Gy equivalent; OAR, organ at risk; PH-SBRT, photon based Stereotactic body radiation therapy; PT-SBRT, proton based Stereotactic body radiation therapy; PTV, planning target volume; SD, standard deviation; V50%/VTotal, volume receiving 50% of the prescription dose/total volume receiving the prescription dose.

a Considered statistically significant based on *P*-value < .05.

### Target coverage

Target coverage was comparable between photon and proton plans for both GTV and CTV coverage with no statistical difference in GTV (99.74 vs 99.40, *P* = .24) (Figure 2A) and CTV (99.70 vs 99.62, *P* = .23) (Figure 2B), with >99% of the target receiving 100% of the prescription dose. Whereas 95% (95.43 vs 94.13, *P* < .01) and 90% (95.43 vs 94.13, *P* < .01) of PTV were better covered with proton than photon plans (Figure 2C).

### Organs at risk comparison

The DVH was significantly improved for proton plans as follows: for small bowel from the V5 Gy to V25 Gy and maximum dose (10.47, 20.22, *P* = .02) (Figure 2D), similarly for large bowel from the V5 Gy to V25 Gy and maximum dose (16.16, 23.90, *P* = .03) (Figure 2E), all volumes of the uninvolved ipsilateral kidney (ipsilateral kidney – CTV) V5 Gy (0.35, 0.61, *P* = .00), V10 Gy (0.29, 0.47, *P* = .00), V15 Gy (0.24, 0.36, *P* = .00), V20 Gy (0.21, 0.27, *P* = .00), V25 Gy (0.18, 0.21, *P* = .00), V30 Gy (0.15, 0.16, *P* = .01) and maximum dose (0.90, 14.61, *P* = .02) (Figure 2F); the heart maximum dose (0.01, 1.10, *P* = .03) (Figure S2A), lungs maximum dose (0.0, 0.18, *P* = .02) (Figure S2B), esophagus maximum dose (0.01, 1.11, *P* = .01) (Figure S2C), stomach maximum dose (1.72, 8.55, *P* = .00) (Figure S2D), duodenum maximum dose (6.15, 12.22, *P* = .02) (Figure S2E), ipsilateral ureter maximum dose (15.54, 27.18, *P* = .04) (Figure S2I), and spinal canal maximum dose (2.21, 9.00, *P* = .00) (Figure S2J). The maximum dose of the proton plans was significantly higher than the photons plans (52.51, 52.35, *P* = .00) (Figure S2L). Integral dose (body – CTV) was significantly lower with intensity modulated proton therapy than with VMAT (0.06, 1.7, *P* = .00) (Figure S2M). The mean conformity index of the proton plans was 0.90 and photon plans was 0.94.

#### Assessment of 42 Gy in three fractions of 14 Gy with Dmax 142%

**Figure 3:**
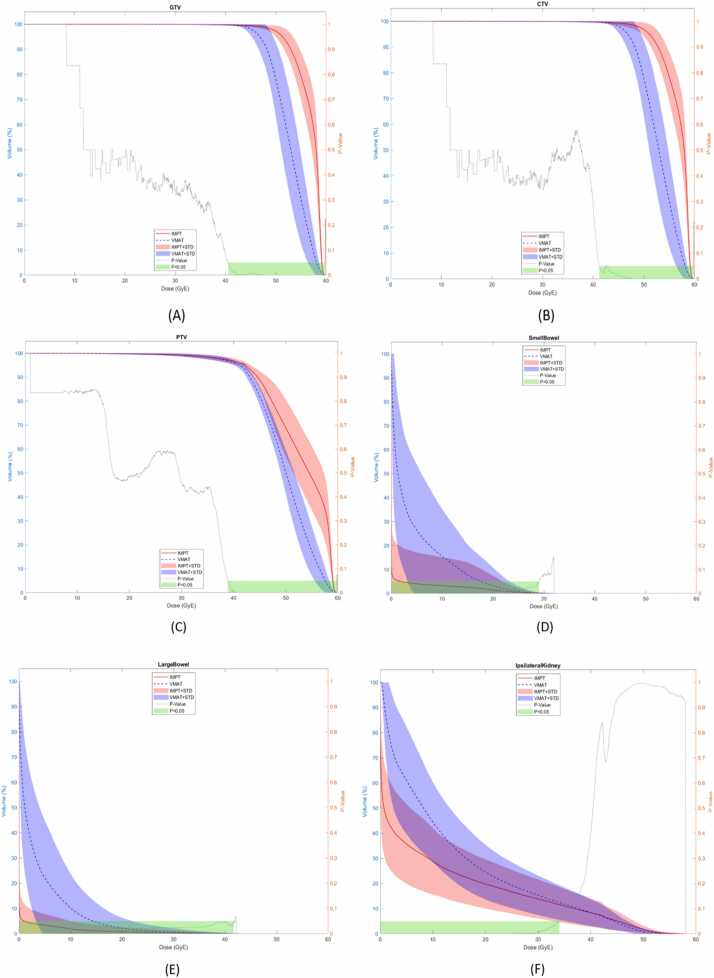
(A–F) (maximum dose 142%): Doses to the targets and organs at risk (OAR) shown over the entire dose–volume histogram comparing intensity-modulated proton therapy (IMPT—red) and Volumetric Modulated Arc Therapy (VMAT—blue), including GTV (A), CTV (B), PTV (C) small bowel (D), large bowel (E), ipsilateral kidney – CTV (F). Abbreviations: CTV, clinical target volume; GTV, gross tumor volume; IMPT, intensity modulated proton therapy; PTV, planning target volume.

The Comparative DVHs for targets and OARs are shown in (Figure 3) and (Supplementary S3). Overall proton therapy allowed for further dose escalation while meeting target coverage goals and OAR constraints in Table 2.

### Target coverage

There was no statistical difference in GTV (99.80, 99.63, *P* = .09) (Figure 3A) and CTV (99.72, 99.63, *P* = .20) (Figure 3B) coverage between (PT-SBRT) and (PH-SBRT) plans, with >99% of the target receiving 100% of the prescription dose. Whereas 95% (95.54, 93.80, *P* = .00) and 90% (95.54, 93.80, *P* = .00) of PTV continued to be better covered with proton than photon plans (Figure 3C).

### Organs at risk comparison

Protons plans were able to maintain improvement of DVH as compared to photon plans including: V5 Gy to V25 Gy and maximum dose of the small bowel (10.13, 20.07, *P* = .00) (Figure 3D), V5 Gy to V25 Gy and maximum dose of the large bowel maximum dose (16.03, 24.23, *P* = .01) (Figure 3E), all volumes of ipsilateral kidney – CTV; V5 Gy (0.36, 0.61, *P* = .00), V10 Gy (0.29, 0.44, *P* = .00), V15 Gy (0.24, 0.32, *P* = .00), V20 Gy (0.20, 0.25, *P* = .00), V25 Gy (0.17, 0.19, *P* = .00), V30 Gy (0.14, 0.15, *P* = .01) and maximum dose (0.81, 12.44, *P* = .04) (Figure 3F), heart maximum dose (0.01, 0.10, *P* = .02) (Figure S3A), lungs maximum dose (0.00, 0.14, *P* = .02) (Figure S3B), esophagus maximum dose (0.00, 1.01, *P* = .01) (Figure S3C), stomach maximum dose (1.72, 8.55, *P* = .00) (Figure S3D), duodenum maximum dose (5.70, 11.70, *P* = .01) (Figure S3E), ipsilateral ureter maximum dose (15.40, 24.42, *P* = .04) (Figure S3I), and spinal canal maximum dose (2.30, 9.20, *P* = .00) (Figure S3J).

Similarly, Integral dose (body – CTV) was significantly lower with PT-SBRT than with PH-SBRT (0.61, 16.70, *P* = .00) (Figure S3M). The mean conformity index of the proton and photon plans remains the same (0.90) and (0.94), respectively.

In addition, dose escalated proton plans allowed for more improvement of the following dosimetric OAR endpoints: stomach V 10 Gy (0.00,0.04, *P* = .05), liver V 25 Gy (0.01, 0.02, *P* = .00), large bowel V 30 Gy (0.00, 0.01, *P* = .02), V 35 Gy (0.28, 0.16, *P* = .02), ipsilateral kidney cortex maximum dose (12.70, 17.40, *P* = .00). The V50%/VTotal (volume receiving 50% of the prescription dose/total volume receiving the prescription dose) was significantly lower with PT-SBRT than with PH-SBRT for both maximum dose of 125% (25.41, 19.97, *P* = .00) and 142% (23.33, 19.18, *P* = .00).

## Discussion

The results from our study show that PT-SBRT provides superior PTV coverage while significantly reducing doses to OARs compared to PH-SBRT. We found that several dosimetric endpoints were significantly lower with PT-SBRT, including maximum doses to heart, lungs, esophagus, stomach, duodenum, small and large bowel, spinal canal, body and the whole-body integral dose, volumes of small and large intestine receiving doses between 5 and 25 GyE and all volumes of ipsilateral kidney and ureter.

Notably, with creation of more heterogenous plans with higher peak tumor dose of 142%, PT-SBRT plans were able to spare more OAR including all volumes of large bowel and ipsilateral kidney cortex without losing the plan conformity.

The conformity indices of proton and photon plans were consistent with both 125% and 142% Dmax plans indicating that the treatment plan continues to deliver the prescribed dose to the target volume with a similar level of conformity.

While Baydoun et al.^12^ reported a comparative dosimetric analysis of Cyberknife, VMAT and proton plans of SBRT for RCC. They found that both VMAT and proton plans provided overall an equivalent or superior coverage to the target volume, while limiting dose to the nearby structures. While our findings are overall consistent with Baydoun’s study, we further report on the improved target coverage, dose heterogeneity, normal tissue sparing, and plan conformity while dose escalating with PT-SBRT.

The impact on renal function is an important factor to consider when determining if SBRT is appropriate for treating RCC. In the FASTRACK II study, the authors reported on the dose-effect relationship of SBRT with posttreatment renal function. They showed that the major loss in renal function occurred in high dose regions (>100 Gy BED3). The ipsilateral kidney eGFR decreased notably from baseline by 42% and 39% in the single fraction cohort and by 45% and 62% in the multi-fraction cohort, at 12 and 24 months, respectively.^13^ The ratio of ipsilateral kidney V50%/Vtot (volume receiving 50% of the dose/total volume) was correlated with GFR loss at 12 and 24 months for both the single and multi-fraction cohorts, indicating that the larger volume of kidney encompassed within the high dose region, the more decline in renal function would result. They recommended reducing the volume receiving 50% of the dose (irradiated volume) to minimize loss of renal function following treatment.^13^ In our study, we found that the irradiated volume (V50%) was significantly lower with protons than conventional photon plans highlighting the potential benefit of PT-SBRT to reduce the GFR loss.

In addition, despite the excellent results of FASTRACK II, it is noteworthy to mention that the study cohort included patients with a good baseline kidney function (mean eGFR was 61.1 mL/min per 1.73 m^2^, 95% CI 56.5–65.6) and excluded cases with direct contact of the target tumor with bowel.^9^, ^14^ Therefore, proton therapy with its unique properties may allow for expanded criteria and a promising option in certain challenging circumstances including patients with poor or fair baseline kidney function, a solitary kidney, or large tumors close to bowel.

Several lung SBRT studies have demonstrated that a higher target Dmax was correlated with optimal local control, which can translate into survival benefit.^15^, ^16^, ^17^, ^18^ In our study, higher intensity PT-SBRT plans with escalation of Dmax resulted in more sparing of nearby critical structures like the ipsilateral renal cortex and large bowel without losing the plan conformity. This may represent another advantage to use PT-SBRT for patients with primary RCC.

With the increasingly robust data showing the benefits of conventional SBRT for treatment of primary RCC and the preponderance of evidence showing the same of proton therapy over photon therapy for hypofractionated treatment in multiple disease sites,^19^ suggest that it is timely to explore the potential role of proton based SBRT in treatment of RCC.

This analysis highlights the potential of PT-SBRT to improve target coverage while reducing doses to healthy tissues particularly the ipsilateral kidney, small and large bowel. To validate our results, our institution is currently running SPARE study (NCT06376669), a single-arm Phase II trial evaluating the use of proton based SBRT for treatment of primary RCC.^20^

### Limitations

While it was beyond the scope of this dosimetric analysis, an important consideration is the type and quality of daily image guidance and motion management technique employed as these may differ across modalities and influence treatment selection. All patients in this cohort were treated with cone beam computed tomography (CBCT) for PH-SBRT and both CBCT and kV for PT-SBRT. Fiducial marker placement and utilization was used to aid in PT delivery when feasible given the inferior CBCT image quality with PT delivery. For PT patients, a baseline and mid-course quality assurance or verification CT scan was obtained routinely to assess any need for plan adaption during PT delivery. As noted in the Methods section, all PT plan optimization combined expansion and robust optimization with 3.5% range uncertainty and 0.5 cm setup uncertainty. Nonetheless, for the purposes of the dosimetric comparison of the nominal plan, robustly optimized PT plans still showed better sparing of OARs compared to PH-SBRT plans.

This study is also limited by the inherent bias of its retrospective design. Another limitation is the lack of correlative toxicity and quality-of-life outcomes to pair with our dosimetric analysis. Therefore, we are planning a follow-up study using normal tissue complication probability (NTCP) calculations to further investigate whether the dosimetric differences shown herein may correlate with clinical outcomes.

## Conclusion

PT-SBRT improved target coverage while reducing dose to the involved kidney and adjacent OARs for patients with primary RCC. Protons allow delivery of maximum peak dose escalated plans without exceeding the normal tissues limit and have the potential to better preserve renal function. Future prospective studies are warranted to validate these dosimetric findings and potential clinical benefits in the management of RCC.

## Ethics approval and consent for publication

The ethical review and approval were waived for this study by the Johns Hopkins Institutional Review Board (IRB) due to the retrospective nature of the study.

## Funding source

This work is supported through the Robert L. Sloan Fund for Cancer Research, awarded from the Albert L. Tucker and Elizabeth T. Tucker Foundation and Sibley Memorial Hospital Foundation.

## CRediT authorship contribution statement

Conceptualization: **Sherif G Shaaban and Curtiland Deville Jr.**. Formal analysis: **Hao Chen and Anh Tran**. Funding acquisition: **Sherif G Shaaban**. Methodology: **Sherif G Shaaban**. Supervision: **Curtiland Deville Jr.**. Writing – original draft: **Sherif G Shaaban**. Writing – review & editing: **Sherif G Shaaban, Aditya Halthore, Rachit Kumar, Michael Goldstein, Daniel Song, Stephen Greco, Heng Li and Curtiland Deville Jr**.

## Declaration of Competing Interest

The authors declare that they have no known competing financial interests or personal relationships that could have appeared to influence the work reported in this paper.

## Data Availability

The data presented in this study are available on request from the corresponding author. The data is not publicly available due to privacy restrictions.

## Footnotes

Appendix A Supplementary data associated with this article can be found in the online version at doi:10.1016/j.ijpt.2025.101210.
