# Supplementary material for: In Silico Comparison of Photon Versus Proton Based Stereotactic Body Radiotherapy With Increasing Maximum Peak Dose for Treatment of Primary Renal Cell Carcinoma
Source: Int J Part Ther. 2025 Oct 25;18:101210. doi: 10.1016/j.ijpt.2025.101210 (PMC12634305; doi:10.1016/j.ijpt.2025.101210)

**In Silico Comparison of Photon Versus Proton Based Stereotactic Body Radiotherapy (SBRT) with Increasing Dose Heterogeneity for Treatment of Primary Renal Cell Carcinoma (RCC)**

Table of Contents:

Item S1- All dosimetric endpoints of target coverage and OAR doses comparing VMAT and IMPT ……………………………………………………………………………………………...2

Item S2- Doses to organ at risk (OAR) shown over the entire dose–volume histogram comparing intensity-modulated proton therapy (IMPT) and Volumetric Modulated Arc Therapy (VMAT) with Dmax of 125%.........................................................................................................................5

Item S3- Doses to organ at risk (OAR) shown over the entire dose–volume histogram comparing intensity-modulated proton therapy (IMPT) and Volumetric Modulated Arc Therapy (VMAT) with Dmax 142%.............................................................................................................................8

**Table S1.** All dosimetric endpoints of target coverage and OAR doses comparing proton based SBRT (PT-SBRT) and photon based

SBRT (PH-SBRT)

| Target/OAR | Dosimetric Endpoint | Maximum Dose 125% | | | Maximum Dose 142% | | |
| --- | --- | --- | --- | --- | --- | --- | --- |
|  |  | PT-SBRT | PH-SBRT |  | PT-SBRT | PH-SBRT |  |
|  |  | Mean ± SD | Mean ± SD | P- value | Mean ± SD | Mean ± SD | P- value |
| GTV | V100% > 99% (%) | 99.74 ± 0.32 | 99.70 ± 0.40 | 0.24 | 99.80 ± 0.30 | 99.63 ± 0.40 | 0.09 |
| CTV | V100% > 99% (%) | 99.70 ± 0.35 | 99.62 ± 0.40 | 0.23 | 99.72 ± 0.33 | 99.63 ± 0.40 | 0.20 |
| PTV | V100% > 95% (%)  V100% > 90% (%) | 95.43 ± 0.90  95.43 ± 0.90 | 94.13 ± 1.81  94.13 ± 1.81 | 0.00^⁎^  0.00^⁎^ | 95.54 ± 1.10  95.54 ± 1.10 | 93.80 ± 1.80  93.80 ± 1.80 | 0.00^⁎^  0.00^⁎^ |
| PTV | Conformity Index (CI) | 0.90 ± 0.04 | 0.94 ± 0.04 | 0.01^⁎^ | 0.90 ± 0.05 | 0.95 ± 0.03 | 0.01^⁎^ |
| Heart | Max Dose (GyE) | 0.01 ± 0.02 | 0.10 ± 2.18 | 0.02^⁎^ | 0.01 ± 0.02 | 0.10 ± 2.18 | 0.02^⁎^ |
| Lungs | Max Dose (GyE) | 0.00 ± 0.01 | 0.18 ± 0.40 | 0.02^⁎^ | 0.00 ± 0.01 | 0.14 ± 0.30 | 0.01^⁎^ |
| Esophagus | Max Dose (GyE) | 0.00 ± 0.02 | 1.01 ± 2.11 | 0.01^⁎^ | 0.00 ± 0.02 | 1.01 ± 2.11 | 0.01^⁎^ |
| Stomach | V5 GyE (cc)  V10 GyE (cc)  V15 GyE (cc)  V20 GyE (cc)  V25 GyE (cc)  V30 GyE (cc)  V35 GyE (cc)  Max Dose (GyE) | 0.00 ± 0.01  0.00 ± 0.01  0.00 ± 0.00  0.00 ± 0.00  0.00 ± 0.00  0.00 ± 0.00  0.00 ± 0.00  1.72 ± 7.62 | 0.15 ± 0.29  0.04 ± 0.12  0.02 ± 0.08  0.01 ± 0.03  0.00 ± 0.00  0.00 ± 0.00  0.00 ±0.00  12.22 ± 10.90 | 0.01^⁎^  0.07  0.16  0.16  NA  NA  NA  0.00^⁎^ | 0.00 ± 0.01  0.00 ± 0.01  0.00 ± 0.00  0.00 ± 0.00  0.00 ± 0.00  0.00 ± 0.00  0.00 ± 0.00  1.70 ± 7.53 | 0.14 ± 0.27  0.04 ± 0.11  0.02 ± 0.07  0.01 ± 0.03  0.00 ± 0.00  0.00 ± 0.00  0.00 ± 0.00  8.13 ± 8.65 | 0.01^⁎^  0.04^⁎^  0.16  0.16  NA  NA  NA  0.00^⁎^ |
| Duodenum | V5 GyE (cc)  V10 GyE (cc)  V15 GyE (cc)  V20 GyE (cc)  V25 GyE (cc)  V30 GyE (cc)  V35 GyE (cc)  Max Dose (GyE) | 0.05 ± 0.12  0.02 ± 0.06  0.01 ± 0.02  0.00 ± 0.00  0.00 ± 0.00  0.00 ± 0.00  0.00 ± 0.00  6.15 ± 9.87 | 0.42 ± 0.44  0.30 ± 0.38  0.14 ± 0.22  0.03 ± 0.06  0.00 ± 0.00  0.00 ± 0.00  0.00 ± 0.00  12.22 ± 10.90 | 0.00^⁎^  0.00^⁎^  0.00^⁎^  0.02^⁎^  NA  NA  NA  0.02^⁎^ | 0.04 ± 0.12  0.02 ± 0.06  0.00 ± 0.02  0.00 ± 0.00  0.00 ± 0.00  0.00 ± 0.00  5.70 ± 9.08  5.70 ± 9.08 | 0.04 ± 0.12  0.02 ± 0.06  0.00 ± 0.02  0.00 ± 0.00  0.00 ± 0.00  0.00 ± 0.00  0.00 ± 0.00  11.70 ± 10.28 | 0.00^⁎^  0.00^⁎^  0.00^⁎^  0.02^⁎^  NA  NA  NA  0.01^⁎^ |
| Liver | V5 GyE (cc)  V10 GyE (cc)  V15 GyE (cc)  V20 GyE (cc)  V25 GyE (cc)  V30 GyE (cc)  V35 GyE (cc)  D700cc ≤ 21 (GyE) | 0.05 ± 0.07  0.03 ± 0.05  0.02 ± 0.04  0.02 ± 0.03  0.01 ± 0.02  0.01 ± 0.01  0.01 ± 0.01  0.16 ± 0.40 | 0.15 ± 0.22  0.09 ± 0.14  0.06 ± 0.10  0.04 ± 0.07  0.02 ± 0.05  0.01 ± 0.04  0.01 ± 0.01  0.70 ± 0.90 | 0.00^⁎^  0.00^⁎^  0.01^⁎^  0.03^⁎^  0.08  0.15  0.37  0.01^⁎^ | 0.05 ± 0.07  0.03 ± 0.05  0.02 ± 0.03  0.02 ± 0.03  0.01 ± 0.02  0.01 ± 0.01  0.01 ± 0.01  1.61 ± 3.80 | 0.14 ± 0.21  0.09 ± 0.14  0.06 ± 0.10  0.03 ± 0.06  0.02 ± 0.04  0.01 ± 0.03  0.01 ± 0.01  6.65 ± 8.11 | 0.00^⁎^  0.00^⁎^  0.01^⁎^  0.02^⁎^  0.04^⁎^  0.12  0.67  0.01^⁎^ |
| Small Bowel | V5 GyE (cc)  V10 GyE (cc)  V15 GyE (cc)  V20 GyE (cc)  V25 GyE (cc)  V30 GyE (cc)  V35 GyE (cc)  Max Dose (GyE) | 0.04 ± 0.13  0.03 ± 0.12  0.03 ± 0.10  0.01 ± 0.05  0.00 ± 0.02  0.00 ± 0.00  0.00 ± 0.00  10.47 ± 13.64 | 0.26 ± 0.27  0.15 ± 0.19  0.08 ± 0.13  0.03 ± 0.08  0.01 ± 0.02  0.00 ± 0.00  0.00 ± 0.00  20.22 ± 12.90 | 0.00^⁎^  0.00^⁎^  0.00^⁎^  0.00^⁎^  0.01^⁎^  NA  NA  0.00^⁎^ | 0.04 ± 0.13  0.03 ± 0.12  0.03 ± 0.10  0.01 ± 0.06  0.00 ± 0.02  0.00 ± 0.00  0.00 ± 0.00  10.13 ± 13.50 | 0.26 ± 0.27  0.15 ± 0.20  0.08 ± 0.13  0.03 ± 0.08  0.01 ± 0.02  0.00 ± 0.00  0.00 ± 0.00  20.07 ± 11.61 | 0.00^⁎^  0.00^⁎^  0.00^⁎^  0.00^⁎^  0.01^⁎^  NA  NA  0.00^⁎^ |
| Large Bowel | V5 GyE (cc)  V10 GyE (cc)  V15 GyE (cc)  V20 GyE (cc)  V25 GyE (cc)  V30 GyE (cc)  V35 GyE (cc)  Max Dose (GyE) | 0.03 ± 0.05  0.02 ± 0.03  0.01 ± 0.02  0.01 ± 0.02  0.00 ± 0.01  0.00 ± 0.01  0.00 ± 0.01  16.16 ± 15.50 | 0.22 ± 0.23  0.10 ± 0.13  0.04 ± 0.07  0.02 ± 0.05  0.01 ± 0.01  0.01 ± 0.02  0.00 ± 0.01  23.90 ± 11.54 | 0.00^⁎^  0.00^⁎^  0.00^⁎^  0.02^⁎^  0.04^⁎^  0.07  0.09  0.03^⁎^ | 0.03 ± 0.04  0.02 ± 0.03  0.01 ± 0.02  0.01 ± 0.02  0.00 ± 0.01  0.00 ± 0.01  0.28 ± 0.01  16.03 ± 15.40 | 0.22 ± 0.23  0.10 ± 0.14  0.05 ± 0.08  0.02 ± 0.04  0.01 ± 0.02  0.01 ± 0.02  0.16 ± 0.01  24.23 ± 11.80 | 0.00^⁎^  0.00^⁎^  0.00^⁎^  0.02^⁎^  0.02^⁎^  0.02^⁎^  0.02^⁎^  0.01^⁎^ |
| Ipsilateral Kidney - CTV | V5 GyE (cc)  V10 GyE (cc)  V15 GyE (cc)  V20 GyE (cc)  V25 GyE (cc)  V30 GyE (cc)  V35 GyE (cc)  Max Dose (GyE) | 0.35 ± 0.15  0.29 ± 0.13  0.24 ± 0.11  0.21 ± 0.10  0.18 ± 0.09  0.15 ± 0.08  0.12 ± 0.07  0.90 ± 11.30 | 0.61 ± 0.20  0.47 ± 0.17  0.36 ± 0.14  0.27 ± 0.12  0.21 ± 0.10  0.16 ± 0.08  0.13 ± 0.07  14.61 ± 0.72 | 0.00^⁎^  0.00^⁎^  0.00^⁎^  0.00^⁎^  0.00^⁎^  0.01^⁎^  0.08  0.02^⁎^ | 0.36 ± 0.16  0.29 ± 0.13  0.24 ± 0.11  0.20 ± 0.10  0.17 ± 0.09  0.14 ± 0.08  0.12 ± 0.07  0.81 ± 11.20 | 0.61 ± 0.21  0.44 ± 0.16  0.32 ± 0.13  0.25 ± 0.11  0.19 ± 0.09  0.15 ± 0.08  0.13 ± 0.07  12.44 ± 0.74 | 0.00^⁎^  0.00^⁎^  0.00^⁎^  0.00^⁎^  0.00^⁎^  0.00^⁎^  0.06  0.04^⁎^ |
| Ipsilateral Kidney Cortex | V5 GyE (cc)  V10 GyE (cc)  V15 GyE (cc)  V20 GyE (cc)  V25 GyE (cc)  V30 GyE (cc)  V35 GyE (cc)  Max Dose (GyE) | 0.34 ± 0.13  0.28 ± 0.11  0.23 ± 0.10  0.20 ± 0.09  0.17 ± 0.08  0.14 ± 0.08  0.11 ± 0.07  13.25 ± 14.18 | 0.61 ± 0.20  0.47 ± 0.18  0.35 ± 0.16  0.26 ± 0.13  0.20 ± 0.10  0.16 ± 0.08  0.12 ± 0.07  19.70 ± 11.80 | 0.00^⁎^  0.00^⁎^  0.00^⁎^  0.00^⁎^  0.00^⁎^  0.00^⁎^  0.01^⁎^  0.09 | 0.33 ± 0.13  0.27 ± 0.11  0.22 ± 0.10  0.19 ± 0.09  0.16 ± 0.08  0.14 ± 0.08  0.11 ± 0.07  12.70 ± 14.00 | 0.60 ± 0.21  0.44 ± 0.18  0.32 ± 0.16  0.25 ± 0.13  0.20 ± 0.10  0.16 ± 0.09  0.12 ± 0.08  17.40 ± 13.14 | 0.00^⁎^  0.00^⁎^  0.00^⁎^  0.00^⁎^  0.00^⁎^  0.00^⁎^  0.01^⁎^  0.00^⁎^ |
| Ipsilateral Kidney Medulla | V5 GyE (cc)  V10 GyE (cc)  V15 GyE (cc)  V20 GyE (cc)  V25 GyE (cc)  V30 GyE (cc)  V35 GyE (cc)  Max Dose (GyE) | 0.36 ± 0.18  0.29 ± 0.16  0.24 ± 0.16  0.20 ± 0.14  0.17 ± 0.13  0.13 ± 0.11  0.11 ± 0.09  13.57 ± 12.40 | 0.63 ± 0.19  0.34 ± 0.14  0.34 ± 0.14  0.25 ± 0.13  0.19 ± 0.12  0.15 ± 0.10  0.11 ± 0.08  18.06 ± 0.91 | 0.00^⁎^  0.00^⁎^  0.00^⁎^  0.00^⁎^  0.06  0.18  0.40  0.13 | 0.34 ± 0.17  0.27 ± 0.16  0.22 ± 0.15  0.19 ± 0.14  0.16 ± 0.12  0.13 ± 0.11  0.10 ± 0.09  12.60 ± 12.00 | 0.60 ± 0.21  0.43 ± 0.16  0.30 ± 0.13  0.21 ± 0.12  0.16 ± 0.10  0.12 ± 0.08  0.09 ± 0.07  15.30 ± 0.93 | 0.00^⁎^  0.00^⁎^  0.00^⁎^  0.00^⁎^  0.28  0.60  0.74  0.20 |
| Ipsilateral Kidney | V50%/Vtot | 25.41±11.04 | 19.97±9.08 | 0.00^⁎^ | 23.33±10.18 | 19.18±9.33 | 0.00^⁎^ |
| Contralateral Kidney | Max Dose (GyE) | 0.00 ± 0.10 | 1.90 ± 1.50 | 0.16 | 0.00 ± 0.10 | 2.00 ± 1.60 | 0.16 |
| Ipsilateral Ureter | V5 GyE (cc)  V10 GyE (cc)  V15 GyE (cc)  V20 GyE (cc)  V25 GyE (cc)  V30 GyE (cc)  V35 GyE (cc)  Max Dose (GyE) | 0.25 ± 0.35  0.20 ± 0.30  0.16 ± 0.27  0.13 ± 0.23  0.11 ± 0.20  0.07 ± 0.14  0.04 ± 0.08  15.54 ± 19.70 | 0.71 ± 0.42  0.65 ± 0.44  0.55 ± 0.42  0.43 ± 0.41  0.32 ± 0.39  0.25 ± 0.36  0.19 ± 0.33  27.81 ± 16.55 | 0.00^⁎^  0.00^⁎^  0.00^⁎^  0.00^⁎^  0.00^⁎^  0.01^⁎^  0.02^⁎^  0.04^⁎^ | 0.25 ± 0.35  0.20 ± 0.30  0.16 ± 0.25  0.12 ± 0.20  0.09 ± 0.15  0.05 ± 0.10  0.03 ± 0.07  15.40 ± 18.50 | 0.68 ± 0.44  0.63 ± 0.45  0.53 ± 0.43  0.43 ± 0.42  0.34 ± 0.39  0.26 ± 0.36  0.19 ± 0.32  24.42 ± 18.32 | 0.00^⁎^  0.00^⁎^  0.00^⁎^  0.00^⁎^  0.00^⁎^  0.01^⁎^  0.02^⁎^  0.04^⁎^ |
| Skin | V5 GyE (cc)  V10 GyE (cc)  Max Dose (GyE) | 0.10 ± 0.08  0.04 ± 0.06  16.20 ± 5.75 | 0.19 ± 0.18  0.07 ± 0.11  16.65 ± 8.22 | 0.00^⁎^  0.00^⁎^  0.15 | 0.10 ± 0.08  0.05 ± 0.07  17.22 ± 6.01 | 0.19 ± 0.17  0.07 ± 0.10  17.00 ± 8.09 | 0.00^⁎^  0.00^⁎^  0.40 |
| Spinal Canal | Max Dose (GyE) | 2.21 ± 4.60 | 9.00 ± 4.70 | 0.00^⁎^ | 2.30 ± 4.71 | 9.20 ± 4.75 | 0.00^⁎^ |
| Body | Max Dose (GyE) | 52.51 ± 0.20 | 52.35 ± 0.08 | 0.00^⁎^ | 59.30 ± 1.62 | 59.15 ± 0.90 | 0.01^⁎^ |
| Body - CTV | Max Dose (GyE) | 0.06 ± 0.70 | 1.70 ± 1.42 | 0.00^⁎^ | 0.61 ± 0.70 | 16.70 ± 1.35 | 0.00^⁎^ |

**Abbreviations: PT-SBRT: proton based Stereotactic body radiation therapy; PH-SBRT: photon based Stereotactic body**

**radiation therapy; OAR: organ at risk; SD: standard deviation; GTV: gross tumor volume; CTV: clinical target volume; PTV:**

**planning target volume; CI: Conformity Index; GyE: radiobiological Gy equivalent, cc: cubic centimeter. V50%/VTotal (volume**

**receiving 50% of the prescription dose/total volume receiving the prescription dose) * Considered statistically significant based**

**on p-value < 0.05.**

**Figure 2S (A-M) (maximum dose 125%):** Doses to organ at risk (OAR) shown over the entire dose–volume histogram comparing intensity-modulated proton therapy (IMPT—red) and Volumetric Modulated Arc Therapy (VMAT—blue), including Heart (A), Lungs (B), Esophagus (C) Stomach (D), Duodenum (E), Liver (F), Ipsilateral kidney cortex (G), Ipsilateral kidney Medulla (H), Ipsilateral ureter (I), spinal canal (J), skin (K), body (L) and the whole body integral dose (Body -CTV)(M)

**
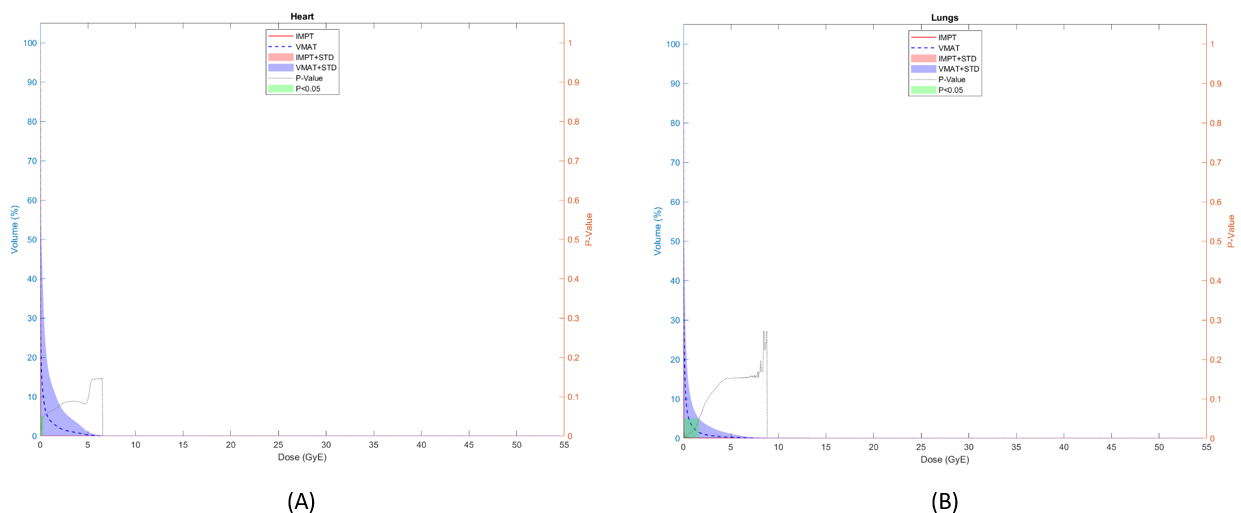
**

**
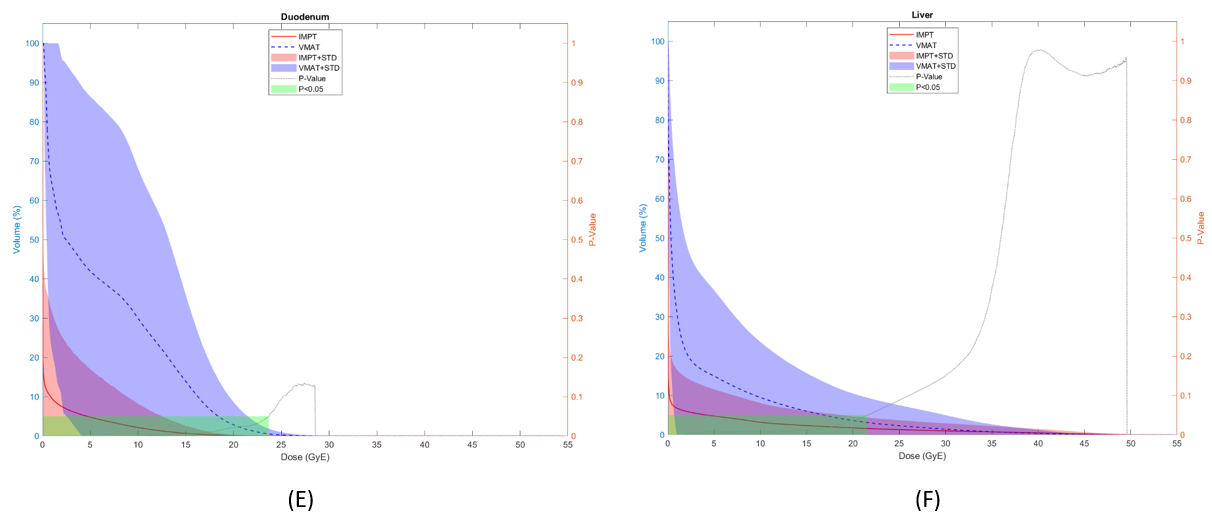

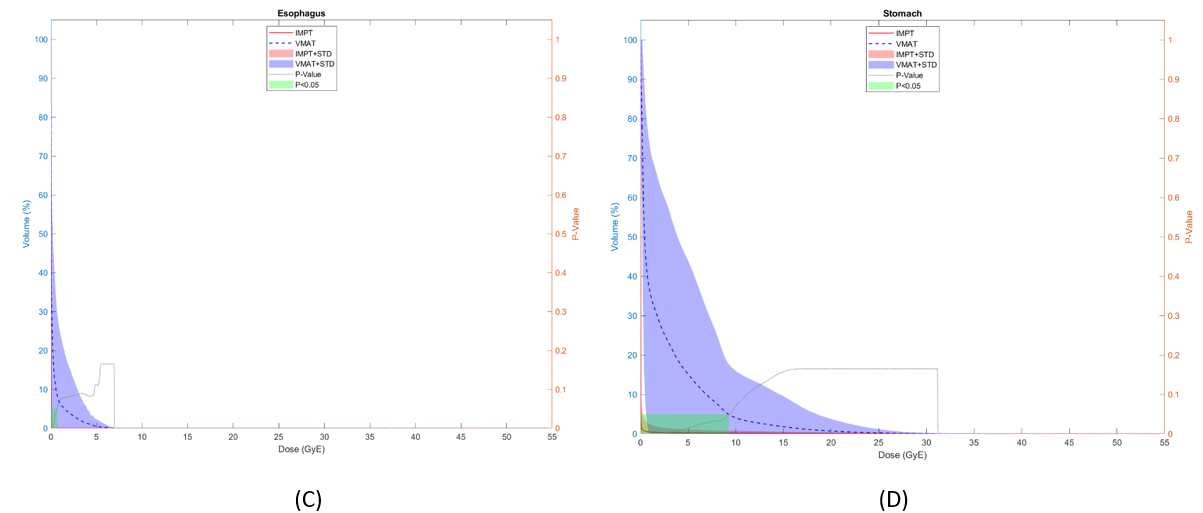
**

**
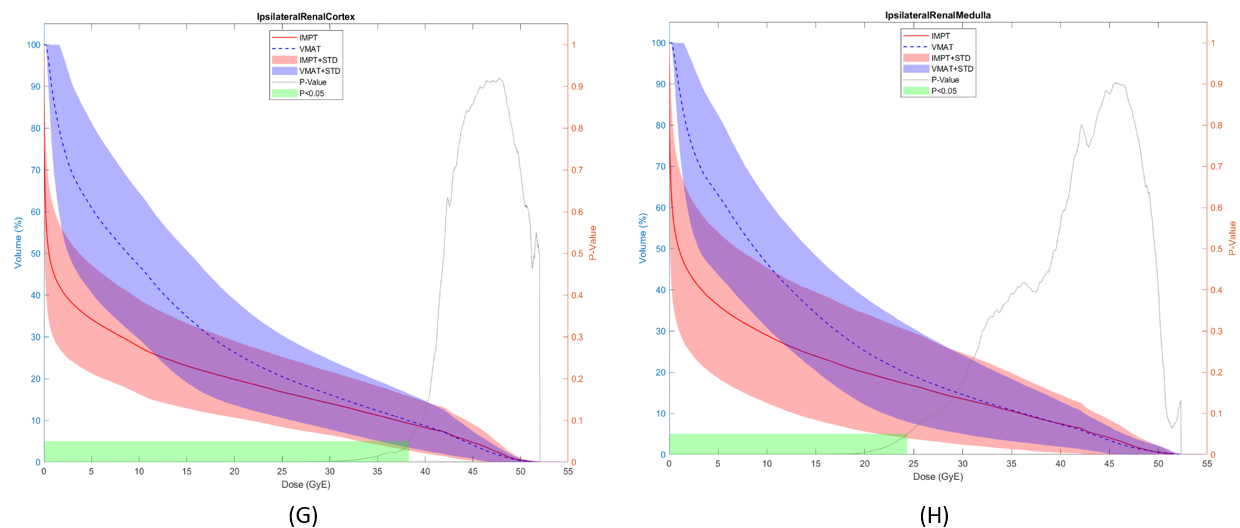
**

**
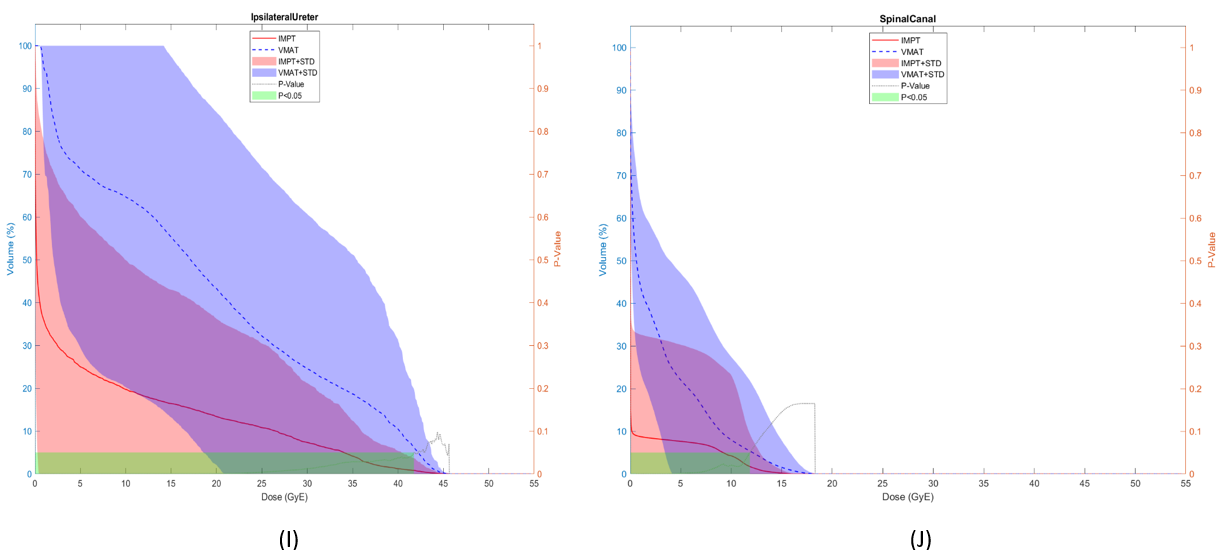
**

**
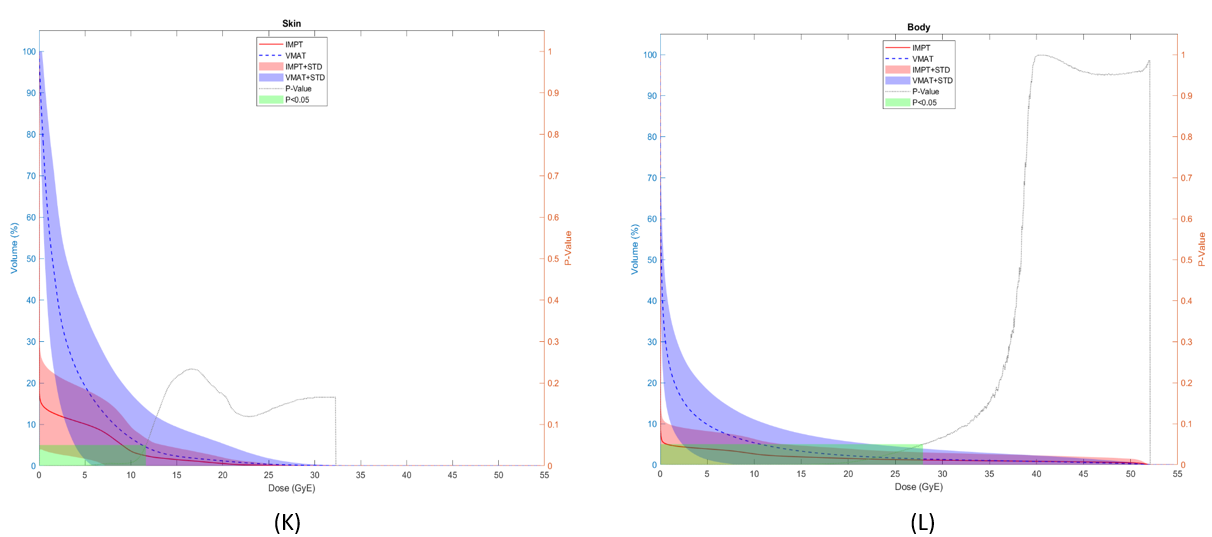
**

**
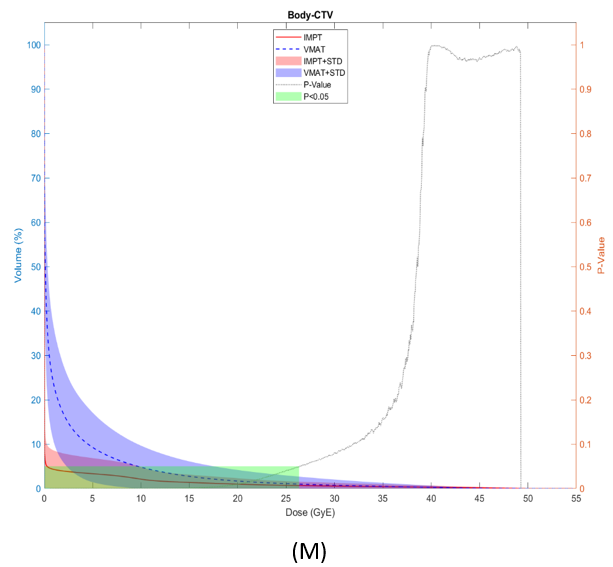
**

**Figure 3S (A-M) (maximum dose 125%):** Doses to organ at risk (OAR) shown over the entire dose–volume histogram comparing intensity-modulated proton therapy (IMPT—red) and Volumetric Modulated Arc Therapy (VMAT—blue), including Heart (A), Lungs (B), Esophagus (C) Stomach (D), Duodenum (E), Liver (F), Ipsilateral kidney cortex (G), Ipsilateral kidney Medulla (H), Ipsilateral ureter (I), spinal canal (J), skin (K), body (L) and the whole body integral dose (Body -CTV) (M)


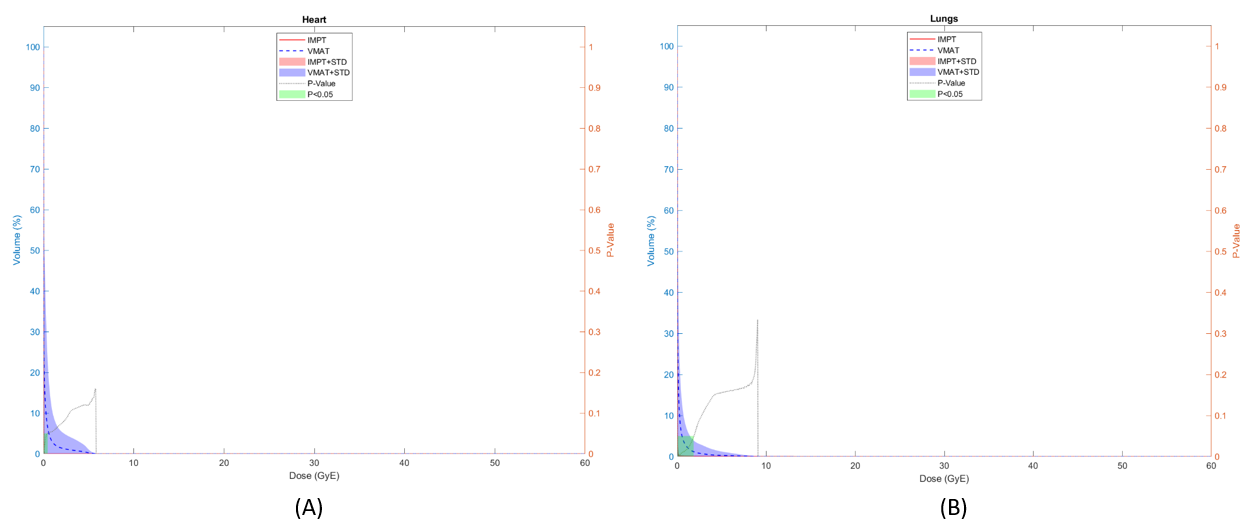


**
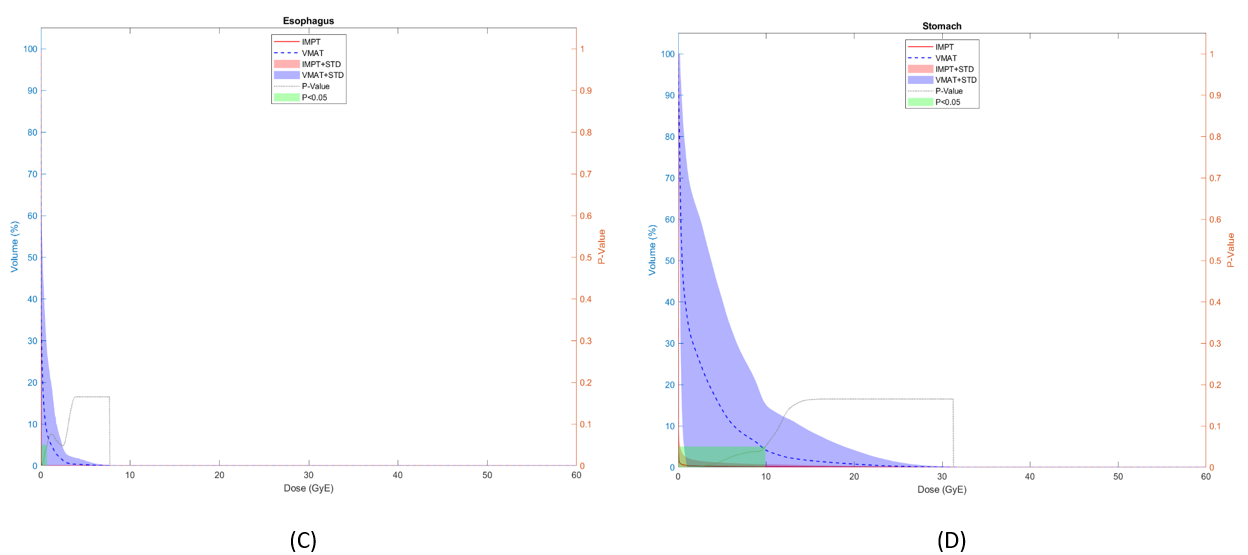
**

**
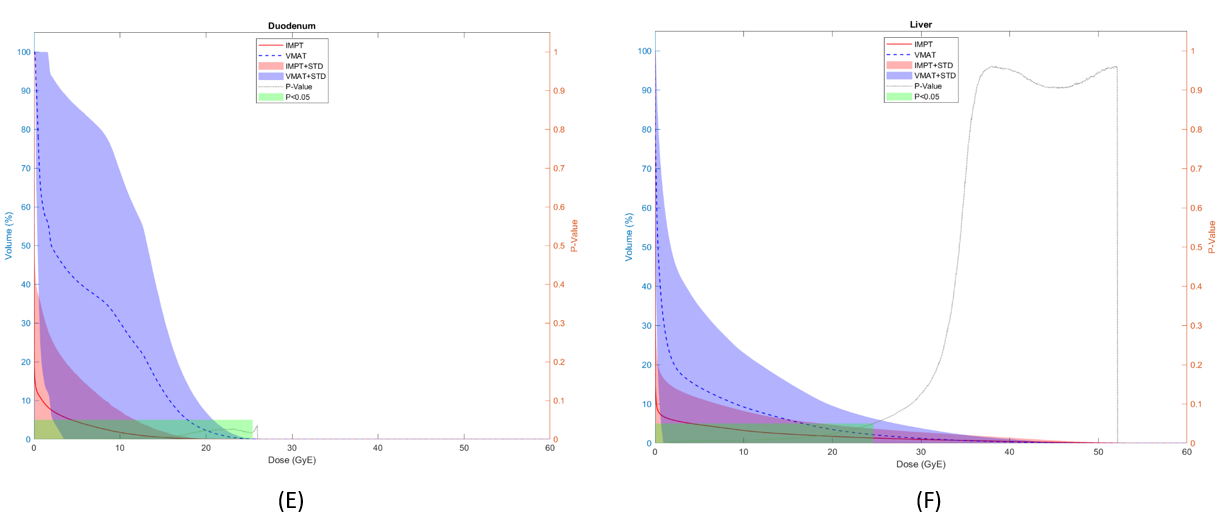
**

**
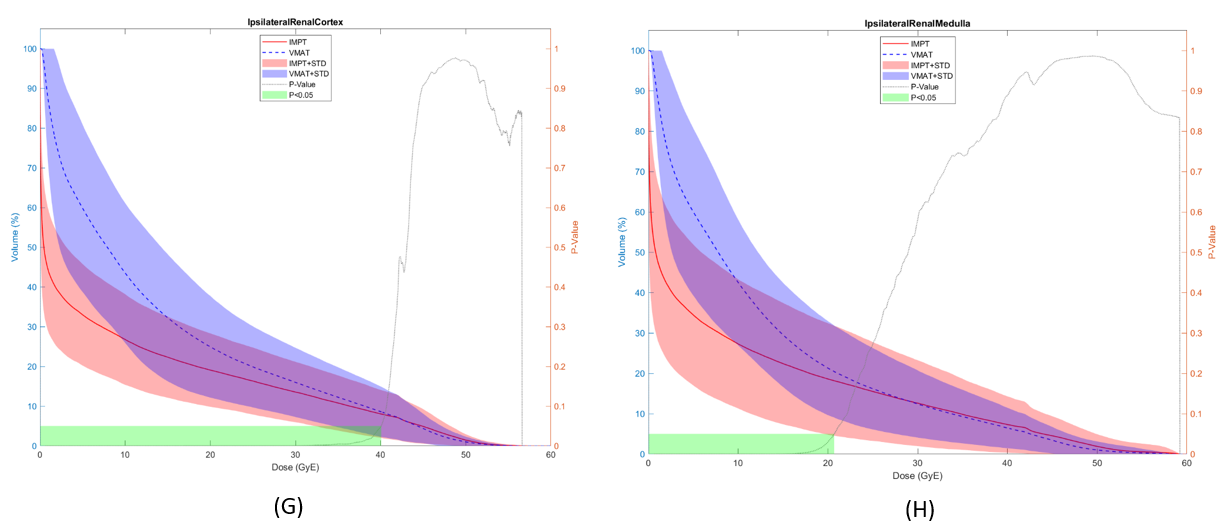
**


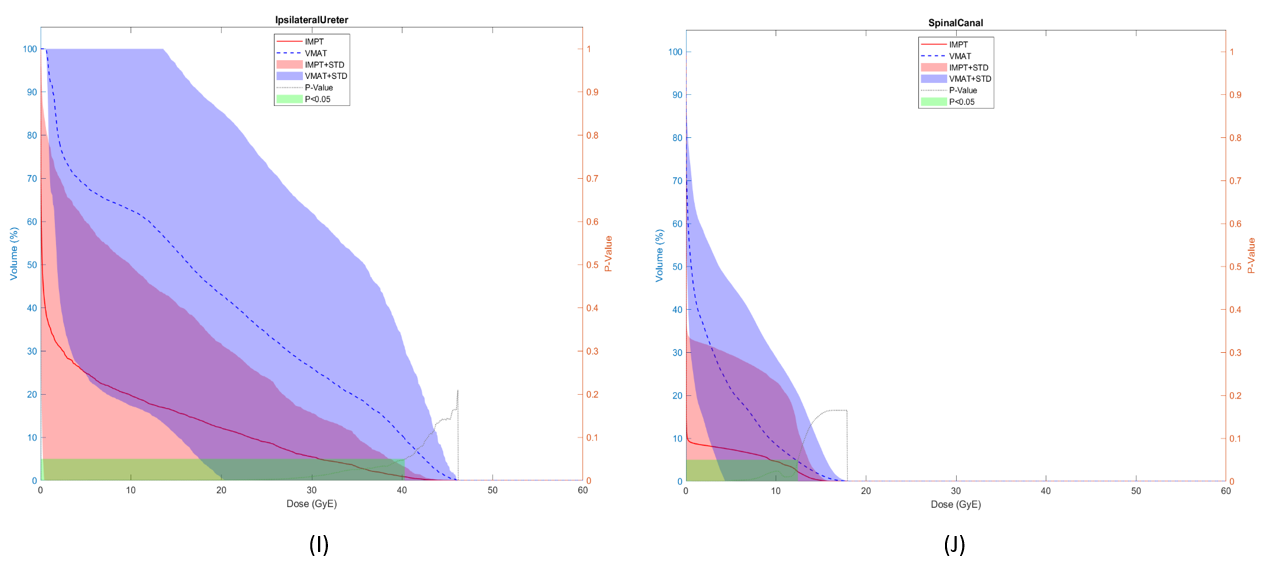


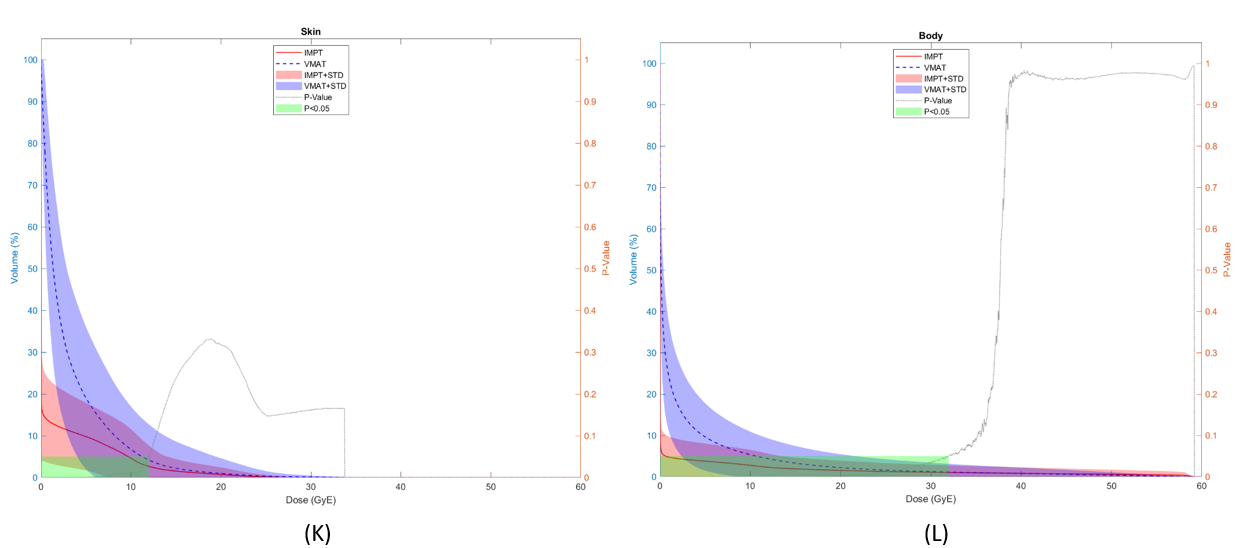


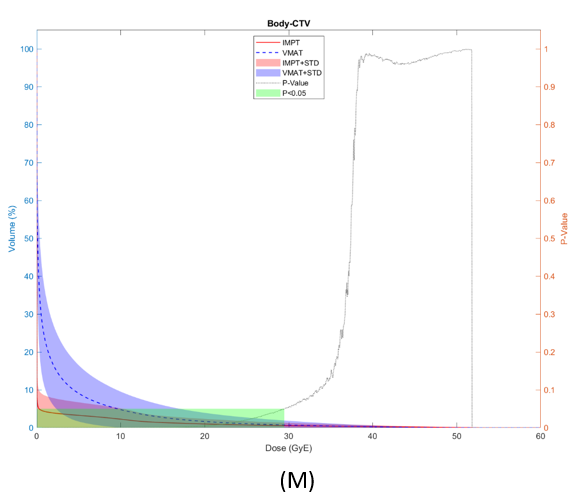

Supplement: Supplementary file 1 — Supplementary material [file mmc1.docx]
